# Introduction: Socialist Humanitarianism

**DOI:** 10.1177/00220094251408474

**Published:** 2026-01-05

**Authors:** Siobhán Hearne

**Affiliations:** 5292 University of Manchester, UK

**Keywords:** humanitarianism, socialism, Cold War, state socialist, NGOs, aid

## Abstract

This special issue places the socialist world at the centre of the modern history of humanitarianism. The contributions bring together a wide range of perspectives to explore the meaning, purpose, and practices of socialist humanitarianism at different scales and across continents. Closely examining practices and discourses from socialist contexts and actors across Africa, Asia, Europe and Latin America expands definitions of humanitarian action, highlights aid flows that upend traditional assumptions about unidirectional Global North-Global South transfer, and proposes alternative chronologies for tracing the development of the international humanitarian sector. This short introduction explores why the socialist world has been sidelined within international historiographies on humanitarianism and argues that placing it at the centre can offer new ways of understanding the evolution of humanitarian ideas and practices across the second half of the twentieth century.

Humanitarian action connected socialists across the world in the context of the Cold War. Socialist aid flowed through multilateral humanitarian organizations and the representatives of socialist states helped to develop the legal frameworks and practices that shaped the international humanitarian sector. Complex long-term medical assistance programmes that were coordinated by national elites in the Global South and the ‘Global Easts’ left a lasting mark upon the built environment and healthcare infrastructure.^1^ Humanitarianism also permeated domestic life within state socialist countries. Through solidarity initiatives, socialist citizens translated their vocal support for national liberation movements and oppressed peoples into tangible material and financial assistance. State socialist regimes called upon voluntary humanitarian organizations to supplement domestic healthcare services by organizing blood donations and providing at-home care services. Beyond state socialist contexts, giving and receiving aid created a complex ecosystem that connected grassroots leftist organizations across continents.

This special issue brings together a diverse range of perspectives to explore the meaning, purpose, and practices of socialist humanitarianism. It deliberately advances a broad definition of socialist humanitarianism as the circulation of aid delivered by individuals, organizations, or governments that identified with socialism. This expansive framing allows us to bring different scales of socialist humanitarianism into conversation, from complex state-led medical assistance programmes to grassroots initiatives that were developed by leftists in Latin America and Western Europe. In this short introduction, I will examine why the socialist world has been sidelined within international historiographies on humanitarianism and argue that placing it at the centre can offer new ways of understanding the evolution of humanitarian ideas and practices across the second half of the twentieth century.

Despite the ubiquity of socialist donors and aid networks, socialist states and actors are chronically underrepresented in historiographies of international humanitarianism. The reasons for this are complex and arguably rooted in the development of the international humanitarian sector in the first half of the twentieth century. In the aftermath of the First World War and the 1917 revolutions that dissolved the Russian Empire, Western relief agencies’ efforts to address widespread displacement, disease, famine, and malnutrition across Eastern Europe and the Near East were often implemented with the dual goals of alleviating suffering and preventing the spread of Bolshevism.^2^ Relief operations deployed in certain regions of the nascent Soviet Union were often critiqued within donor societies for fuelling the development of Bolshevism and supporting the political enemies of the West.^3^ Anti-Bolshevik impulses also deeply shaped the international Red Cross movement's response to the intensification of civil war violence across Europe in the early 1920s.^4^ After the end of the Second World War, the anti-communist thrust of international humanitarian relief efforts accelerated as the wartime coalition of the Allies splintered. Thereafter, Western-led relief and reconstruction assistance to address wartime displacement and destruction were explicitly implemented as a response to the perceived geopolitical and civilizational threats posed by communism.^5^

Historical and contemporary understandings of humanitarianism are also heavily influenced by the experiences of international non-governmental organizations (NGOs) headquartered in the West, including the Red Cross and the United Nations.^6^ Narrow understandings of what humanitarianism is have resulted in the sidelining of ‘humanitarian action not borne of the Northern-dominated and highly institutionalized international regime’, which has meant that relief practices originating in either the Global South or the socialist world have not been fully incorporated into the global history of humanitarianism.^7^ Instead, global histories have tended to take Western perspectives and practices as normative and focus on ‘watershed moments’ that carried enormous significance within North America and Western Europe, but that were less formative elsewhere. In the West, the Biafran War caused an international outpouring of humanitarian activism and sentiment, reshaped the humanitarian sector, and was a key moment of enormous governmental and non-governmental mobilization.^8^ This conflict may have been the catalyst for the West's ‘NGO moment’ during which NGOs became experts in aid provision and desirable partners in the implementation of international political programmes, but the same cannot be said for the socialist world.^9^ Different flashpoints emerge if we look from the vantage point of the ‘Global Easts’. The Korean War (1950–53) was a formative moment for the globalization of socialist humanitarianism. Aid and medical teams from across Central and Eastern Europe, the Soviet Union, China, and Mongolia were dispatched to North Korea, and thousands of Korean child refugees were housed across socialist Eastern Europe.^10^ Decolonization in Africa and the Vietnam War were also important turning points, both for state socialist regimes seeking to showcase themselves as humanitarian donors and for widespread societal humanitarian mobilization. The collapse of socialism in Europe also generated a powerful ‘NGO moment’. Transnational humanitarian aid flowed to formerly state socialist countries and a plethora of informal organizations sprang up to address humanitarian crises, often in collaboration with international organizations and foreign donors.^11^

Definitions of humanitarianism have largely been framed around the fundamental principles of humanity, impartiality, neutrality, and independence, which has also resulted in the exclusion of the socialist world. Innovative scholarship has examined how these theoretically apolitical principles have been (and continue to be) mobilized by NGOs and humanitarian agencies originating in the Global North to perpetuate colonial hierarchies in the Global South.^12^ However, as Elena Fiddian-Qasmiyeh and Julia Pacitto aptly observe, both ‘a strict adherence to the Northern institutional model and the countervailing post-colonial critique of the Northern system’ have rendered other forms of humanitarianism less visible or framed them as deviations from the ‘norm’.^13^ Actors and governments across the socialist world rejected the principle of neutrality and emphasized the inseparability of humanitarianism and politics, particularly anti-imperialism and anti-colonialism.^14^ The same rejection of the neutrality of relief was practiced by prospective recipients of humanitarian aid in the context of the Cold War. In the wake of disaster, certain socialist governments (for example, China and the Soviet Union) refused to accept foreign aid in order to prevent reputational damage domestically and internationally.^15^ The fundamental principle of independence was also largely meaningless in socialist states because organizations who sought to deliver aid required, at the very least, some level of endorsement from the government in order to operate.

Socialist states were also keen to present their humanitarian activities as distinctive from those that originated in the capitalist West. In the context of decolonization, governments in state socialist Europe framed their aid to the Global South as the antithesis of Western philanthropy: an ‘extension of the struggle against fascism and capitalist imperialism’ and a longer-term solution to underdevelopment and dependency.^16^ In communist China, humanitarian action required an entirely different vocabulary because the concept of humanitarianism was so strongly associated with European and US imperialism.^17^ In this special issue, Dongxin Zou explores how Chinese doctors practiced a distinctive form of Maoist humanitarianism in Algeria, wherein they rejected privilege and professional hierarchies and collapsed the boundaries between medical expertise and political consciousness. On the other side of the world in Cuba, humanitarianism has long been framed around the idea of ‘health(care) as political praxis’ and built upon non-hierarchical visions of solidarity that are undergirded by ‘cooperation without political conditionality or material self-interest’.^18^ Since the 1960s, sending Cuban medical brigades overseas has been conceptualized as the antithesis of charitable giving and a method for addressing ‘deeper structures of inequity found within healthcare provision on a global scale’.^19^ Therefore, vocabularies of humanitarian action differed significantly in the socialist world, even if the actual practices of humanitarianism shared common features across geopolitical divides.

Despite its underrepresentation in scholarship, the socialist world was an epicentre of transnational humanitarian intervention and mobilization in the twentieth century. As donors, state socialist states were important links in the global networks of humanitarian aid that flowed from the ‘Second’ to ‘Third’ worlds. Socialist states also received aid and even became centres of international humanitarian intervention in moments of crisis, be it in Tito's Yugoslavia, Ceaușescu's Romania, Mengistu's Ethiopia, or Gorbachev's Soviet Union.^20^ Beyond acting as donors and recipients, socialist states also helped to shape the international humanitarian sector more broadly. After the end of the Second World War, socialist states made major contributions to the development of international humanitarian law, particularly the Geneva Conventions of 1949 and the Additional Protocols to the Geneva Conventions in the 1970s.^21^ Experts from Central and Eastern Europe were instrumental in ensuring the gradual codification of healthcare as a human right in international law throughout the 1940s–1970s.^22^ In the 1970s, non-aligned and socialist countries helped reshape international humanitarian law to include the protection of guerrilla fighters in liberation wars, as well as reform the relationship between national Red Cross and Red Crescent societies and the central bodies of the movement in Geneva.^23^ Socialist states were also enthusiastic participants in high profile international humanitarian programmes that were delivered within the framework of multilateral organizations headquartered in the capitalist West, including the League of Red Cross Societies, the United Nations High Commissioner for Refugees, and the World Health Organization.^24^

Across the twentieth century, humanitarianism provided socialist regimes with a form of legitimization and a method for projecting alternative geopolitical visions on the global stage. In the 1920s, the recognition of the Bolshevik-controlled Russian Red Cross by the International Committee of the Red Cross and the Soviet Union's engagement with international relief agencies lent legitimacy to the fledgling Soviet regime at home and abroad and served to counter negative representations of the new government in the West.^25^ In the 1960s, engagement in humanitarian activities provided national independence movements in the decolonizing world with a method for building legitimacy and mobilizing international support.^26^ During the Cold War, the practice of socialist humanitarianism served to foster imaginaries of a new world order. By providing coordinated and long-term healthcare assistance to the newly-established states that emerged from collapsing European empires, socialist regimes exported their healthcare models to post-colonial contexts, claimed authority within the field of global health, and developed a ‘socialist commonwealth ideal that expanded beyond Europe’.^27^ As Čarna Brković's work on the Yugoslav Red Cross aptly shows, humanitarian initiatives were a form of ‘socialist worldmaking’ that constructed ‘an imaginary of worldwide relations distinct from globalisation and colonialism’.^28^

Socialist humanitarianism was a powerful form of diplomacy that was exercised from both above and below. Visibly supporting national liberation movements through the delivery of aid allowed state socialist regimes to showcase their commitment to anti-imperialism and sharpen the distinction between the West and the East on the global stage.^29^ Outside the framework of state socialist regimes, humanitarian aid was an important manifestation of socialist internationalism during periods of conflict. During the Spanish Civil War and Vietnam War, trade unions, leftist political parties, and activists across Europe, North America, and Asia sent material, medical, and financial aid to Republican Spain and the Democratic Republic of Vietnam.^30^ For citizens in state socialist countries, the act of giving aid – be it money, resources, or blood – was a tangible expression of solidarity with oppressed people and a way to live out the ideals of socialist internationalism.^31^

Beyond the international context, focusing on humanitarianism also offers insight into the numerous ways in which citizens opted in to the task of building socialism, as well as the complex ways in which international politics shaped the social and cultural history of state socialist societies. The ‘home front’ of socialist internationalism was a kaleidoscope of activist solidarity initiatives that were simultaneously coordinated by above and from below.^32^ These initiatives were dynamic spaces that spawned ‘the seeds of both resistance and legitimation’ and where state ideologies were both rigidly enforced and subverted.^33^ Dora Tot's article in this special issue takes readers to the home front of Yugoslav socialist internationalism, where ritualized and state-controlled acts of solidarity provided opportunities for citizens to contribute to the government's humanitarian engagements with national liberation movements and become ‘active participants in socialist worldmaking’. Within state socialist societies, humanitarianism was not only about delivering aid overseas and was also geared towards supplementing domestic healthcare provision through voluntary labour.^34^ Maren Hachmeister's article explores how the Czechoslovak and Polish Red Cross societies served the healthcare system by organizing blood donations. While these socialist Red Cross societies were subordinate to the state, the governments of both countries depended heavily on the voluntary labour of their members to ensure that hospitals and clinics had an adequate supply of blood. Hachmeister shows that through this relationship of dependence, the Red Cross societies in Czechoslovakia and Poland both stabilized and challenged socialist rule in their respective countries.

Placing the socialist world at the centre of the history of humanitarianism also highlights networks of aid distribution that complicate the traditional trajectories of Global North-Global South transfer. Rather than flowing from the West to the ‘rest’, medical expertise, technical assistance, and relief supplies circulated from East-South, East-East, South-East, and South-South throughout the twentieth century. Focusing on Cuba's enormous contributions to the delivery of medical aid globally allows us to not only ‘disrupt the imperial gaze of humanitarianism’, but also to disrupt hierarchies within the socialist world itself.^35^ Cuba sent far greater numbers of doctors, nurses, and teachers on overseas missions than European state socialist regimes, including the self-proclaimed leader of the socialist bloc – the USSR.^36^ Aid also circulated horizontally within and across the socialist world. Mozambique was a hub of both infra-African aid and socialist humanitarianism after independence, as aid and medical personnel arrived from Tanzania, Guinea, Cuba, China, and North Korea, as well as from Western leftist networks.^37^ In the aftermath of the 1988 Armenian earthquake, numerous socialist states that had previously received Soviet aid sent relief supplies to the USSR, including Yugoslavia, Hungary, Vietnam, Romania, North Korea, China, and Mongolia.^38^ Aid also circulated bidirectionally across geopolitical divides and shared experiences motivated socialist citizens to donate to their ‘fraternal’ neighbours.^39^ In this special issue, Sebastian Fonseca's article helps us to reconsider the direction of aid flows through its focus on a transnational grassroots network: the Latin American Social Medicine Association (ALAMES). Against the backdrop of anti-communist terror across Latin America, ALAMES's humanitarianism was a form of epistemic survival for practitioners of social medicine as the group protected persecuted physicians, created institutional safe havens, and disseminated scholarship across borders. As Fonseca argues, the work of ALAMES offers a new perspective on socialist humanitarianism, one that moves beyond the framework of the state and international NGOs to explore grassroots South–South connections.

Humanitarianism also provides a lens for exploring exchange, tensions, and fragmentation within the socialist world itself. The delivery of humanitarian aid from East to South was inflected by intra-socialist hierarchies, as well as a perpetual struggle for resources and influence. Competition and collaboration were core elements of state efforts to export socialist healthcare in the era of decolonization and medical professionals from state socialist countries often worked alongside one another during overseas medical missions.^40^ As Bogdan Iacob shows in this special issue, Romanian officials closely studied the aid programmes of other state socialist governments in order to formulate their own assistance programmes in Sub-Saharan Africa. Similarly, Dongxin Zou's article demonstrates how Chinese doctors in Algeria embraced Maoist humanitarianism as a distinctive offering, which meant rejecting the privileges and remuneration that their counterparts from European socialist countries received and opting to work in remote rural areas. These choices set them apart from the Romanian and Soviet medical missions that are explored in Bogdan Iacob's and my own contribution, which largely focused on enhancing urban healthcare provision in recipient societies, despite the governments of Romania and the USSR publicly espousing the benefits of their medical systems in improving health outcomes for rural populations.

The socialist world also provides a new lens for examining the relationship between donors and recipients of humanitarian aid. Within state socialist Europe, the relationship between socialist donors and recipients was sometimes shaped by a sense of sameness: shared experiences of poverty and scarcity, as well as a perceived sense of mutual dependency.^41^ Beyond the European continent, aid flowing from East to South was built upon a foundation of anticolonial solidarity, but the donor–recipient relationship sometimes replicated colonial dynamics. This paradox was central to European socialist humanitarianism, wherein a sincere commitment to anti-racism and anti-imperialism sat uncomfortably alongside racialized thinking and practice.^42^ Interactions between medical teams from state socialist Eastern Europe and their counterparts in the postcolonial world were often ‘underpinned by the cultural revival of fantasies of Western imperial power’ and discussions of socialist aid drew divisions between the ‘modernity’ of the donor and ‘backwardness’ of the recipient.^43^ The rhetoric and imagery used to mobilize citizens of state socialist contexts to support people across the decolonizing world were also sometimes inflected by imagined civilizational hierarchies.^44^ Recipient societies also projected their own racial hierarchies onto foreign physicians.^45^ In this special issue, Bogdan Iacob explores how ideas about race shaped the donor–recipient relationship between Romanian physicians and the populations that they served in Sub-Saharan Africa. These interactions produced ‘socialist whiteness’: a racial optic premised on ideas about developmental hierarchies, European civilizational superiority, the modernizing potential of socialism, and the pathologization of postcolonial populations as underdeveloped. My own article also examines how civilizational hierarchies permeated Soviet humanitarianism. Not only did publications on Soviet medical missions present local populations as ‘backwards’, but seconded Soviet specialists tended to be recruited from the more ‘developed’ republics of Russia and Ukraine, rather than from the republics of the Caucasus or Central Asia.

Histories of humanitarianism have tended to privilege the perspectives of donors at the expense of recipient governments and societies. Recently, however, a developing body of scholarship has emerged that explores how countries within the Non-Aligned Movement and the socialist world engaged flexibly with an array of state and non-state donors across geopolitical divides.^46^ Two articles in this special issue make important interventions in this regard. First, Alila Brossard Antonielli examines how aid was received in Angola and Mozambique during the Cold War and then remembered in the decades thereafter. In her focus on recipients, Antonielli illuminates how independence movements and other affiliated organizations navigated the complexities of the Cold War aid landscape and leveraged personal connections to secure both medical aid and international recognition. Second, Sebastian Fonseca's exploration of the activities of the Latin American transnational collective ALAMES demonstrates how the boundary between the donor and the recipient could be blurred and even dissolved. Persecuted social medicine practitioners within the network simultaneously received and gave aid to their colleagues in their efforts to resist the leftist ‘epistemicide’ that was enacted as part of Operation Condor in the Southern Cone.

As the scholarship on socialist humanitarianism and this special issue demonstrate, looking from the vantage points of the socialist world offers new ways of considering the modern history of humanitarianism. Closely examining practices and discourses from socialist contexts and actors across Africa, Asia, Europe, and Latin America expands our understanding of humanitarianism beyond the definitions that have been advanced by Northern-dominated multilateral organizations throughout the twentieth century. New geographies of humanitarianism also emerge that upend traditional assumptions about unidirectional Global North–Global South transfer. Decentring humanitarian action that originated in the capitalist West allows us to nuance the chronology of the international humanitarian sector and propose alternative timelines and watershed moments that shaped its development. Taken together, the articles that follow invite readers to consider the socialist world as an important site of humanitarian mobilization and intervention in the twentieth century.

## Notes

1 I borrow this term from the pioneering work of Čarna Brković on the history and anthropology of humanitarianism, who in turn takes inspiration from Jie-Hyun Lim. ‘Global Easts’ is not a synonym for state socialist Europe, but instead a term that connects different places ‘with distinct histories of socialist modernity, that find themselves in this analytical gap between the Global North and the Global South and demand further theorization’, Čarna Brković, *Realigning Humanitarianism in the Balkans: From Cold War Politics to Neoliberal Ethics* (Bloomington: Indiana University Press, 2026), 10. See also Jie-Hyun Lim, *Global Easts: Remembering, Imagining, Mobilizing* (Ithaca: Cornell University Press, 2022).

2 Kimberley A. Lowe, ‘Confrontations with Typhus and Bolshevism: Red Cross Epidemic Relief in Revolutionary Ukraine (1919–1920)’, in Jean-François Fayet, Marie-Luce Desgrandchamps, Marie Cugnet, and Donia Hasler (eds) *The Red Cross Facing the Red Star: Humanitarianism and Communism in the XXth Century* (Geneva: Georg, 2025), 59–74; E. T. Vardağlı, ‘Humanitarian Organizations in the “Greater Near East”: A Shield Against Soviet Russia’, *International History Review,* 45, 4 (2023), 718–33; Tomás Irish, ‘Educating Those Who Matter: Thomas Whittemore, Russian Refugees and the Transnational Organization of Elite Humanitarianism after the First World War’, *European Review of History,* 28, 3 (2021), 441–62; Doina Anca Cretu, *Foreign Aid and State Building in Interwar Romania: In Quest of an Ideal* (Stanford: Stanford University Press, 2024), 55–57; Patrick J. Houlihan, ‘Renovating Christian Charity: Global Catholicism, the Save the Children Fund, and Humanitarianism During The First World War’, *Past & Present,* 250, 1 (2021), 203–41; David S. Foglesong, *America's Secret War Against Bolshevism: U.S. Intervention in the Russian Civil War, 1917–1920* (Chapel Hill: University of North Carolina Press, 1995), chapter 8.

3 Linda Mahood and Vic Satzewich, ‘The Save the Children Fund and the Russian Famine of 1921–23: Claims and Counter-Claims about Feeding “Bolshevik” Children’, *Journal of Historical Sociology,* 22, 1 (2009), 55–56; Rodney Breen, ‘Saving Enemy Children: Save the Children's Russian Relief Operation’, *Disasters*, 18, 3 (1994), 221–37.

4 Robert Gerwarth and Gwendal Piégais, ‘Intra-State Conflicts and the Politics of Aid in Europe, 1917–49: An Introduction’, *Contemporary European History* (2025), 6–7; Kimberley A. Lowe, ‘The Red Cross and the Lessons of the Civil War, 1918–26’, *Contemporary European History* (2025); Jean-François Fayet*,* ‘Humanitaire et Communisme: histoires parallèles et croisées’, in *The Red Cross Facing the Red Star,* 14–15.

5 Joshua Thew, ‘The Muse of Rural Assistance in Greece for Relief and Rehabilitation: The Near East Foundation and United Nations Relief and Rehabilitation Administration, 1944–7’, in Samantha K. Knapton and Katherine Rossy (eds) *Relief and Rehabilitation for a Post-war World: Humanitarian Intervention and the UNRRA* (London: Bloomsbury, 2024), 73–102; David Brydan, ‘Christian Humanitarianism, Refugee Stories, and the Making of the Cold War West’, *Historical Journal,* 66, 3 (2023), 689–714; Francesco Zavatti, ‘“To Be Assisted Secretly”: Catholic Humanitarianism and Romanian Displaced Fascists, 1945–7’, *Journal of Contemporary History* (2025); Panagiotis Karagkounis, ‘Forced Displacement and the Weaponisation of Humanitarian Aid during the Greek Civil War, 1947–50’, *Contemporary European History* (2025).

6 Eleanor Davey, ‘New Players Through Old Lenses: Why History Matters in Engaging with Southern Actors’, *Humanitarian Policy Group Brief,* 48 (July 2012), 2.

7 Julia Pacitto and Elena Fiddian-Qasmiyeh, *Writing the ‘Other’ into Humanitarian Discourse: Framing Theory and Practice in South–South Humanitarian Responses to Forced Displacement,* UN High Commissioner for Refugees New Issues in Refugee Research, Research Paper No. 257 (Geneva: UNHCR, 2013), 3.

8 Michael Barnett, *The Empire of Humanity: A History of Humanitarianism* (Ithaca, 2011); Lasse Heerten, *The Biafran War and Postcolonial Humanitarianism: Spectacles of Suffering* (Cambridge, 2017); Silvia Salvatici, *A History of Humanitarianism, 1755–1989: In the Name of Others* (Manchester, 2019).

9 Kevin O'Sullivan, *The NGO Moment: The Globalization of Compassion from Biafra to Live Aid* (Cambridge, 2021).

10 Bogdan C. Iacob, ‘Paradoxes of Socialist Solidarity: Romanian and Czechoslovak Medical Teams in North Korea and Vietnam (1951–1962)’, *Monde(s),* 20 (2021), 117–40; Dora Vargha, ‘Technical Assistance and Socialist International Health: Hungary, the WHO, and the Korean War’, *History and Technology,* 36 (2020), 400–17; Balázs Szalontai, ‘Small-Power Diplomacy in Northeast Asia: Mongolian–North Korean Relations During the Cold War, 1948–1989’, *North Korean Review,* 12, 2 (2016), 48–49; Kim, Jin-hyouk and Moon, Mira, ‘The Socialist Camp's North Korean Medical Support and Exchange (1945–1958) – Between Learning from the Soviet Union and Independent Course’, *Korean Journal of Medical History,* 28, 1 (2019), 139–90; Bogdan C. Iacob, ‘Health’, in James Mark and Paul Betts (eds) *Socialism Goes Global: The Soviet Union and Eastern Europe in the Age of Decolonisation* (Oxford: Oxford University Press, 2022)*,* 382–86; Péter Apor, ‘The School: Schools as Liminal Spaces – Integrating North Korean Children Within Socialist Eastern Europe, 1951–9’, in Kristin Roth-Ey (ed) *Socialist Internationalism and the Gritty Politics of the Particular: Second-Third World Spaces in the Cold War* (London: Bloomsbury, 2023), 21–38; Nikola Tohma, ‘The Czechoslovak Red Cross and Refugee Children from Greece and North Korea’, in Doina Anca Cretu and Michal Frankl (eds) *Humanitarian Mobilization in Central and Eastern Europe* (Manchester: Manchester University Press, 2025), 115–40; Intaek Hong, ‘North Korean Orphans in Poland Experiences and Legacies of Education in Socialist Internationalism, 1953–1962’, in Damiano Matasci and Raphaëlle Ruppen Coutaz (eds) *Educational Internationalism in the Cold War: Plural Visions, Global Experiences* (Abingdon: Routledge, 2025), 39–54.

11 Laëtitia Atlani-Duault, *Humanitarian Aid in Post-Soviet Countries: An Anthropological Perspective,* translated by Andrew Wilson (Abingdon: Routledge, 2007); Luciana Jinga, ‘(Im)Possible Alliance and its Consequences: the Impact of Transnational Humanitarian Aid and Politics Towards Institutionalized Children in Romania (1990-2007)’, *New Europe College Stefan Odobleja Program Yearbook 2020*–*21* (2021), 153–80.

12 Emily Baughan, *Saving the Children: Humanitarianism, Internationalism, and Empire* (Berkeley: University of California Press, 2021); Poppy Cullen, Steve McCorriston and Andrew Thompson, ‘The “Big Survey”: Decolonisation, Development, and the First Wave of NGO Expansion in Africa after 1945’, *International History Review,* 44, 4 (2022), 721–50; Kevin O'Sullivan, ‘Humanitarian Encounters: Biafra, NGOs and Imaginings of the Third World in Britain and Ireland, 1967–70’, *Journal of Genocide Research,* 16, 2–3 (2014), 299–315; Margaret Tudor, *Blue Helmet Bureaucrats: United Nations Peacekeeping and the Reinvention of Colonialism, 1945–1971* (Cambridge: Cambridge University Press, 2023); Elena Fiddian-Qasmiyeh, ‘Engendering Faith-Based Organizations: Intersections between Religion and Gender in Development and Humanitarian Interventions’, in Anne Coles, Leslie Grey and Janet Momsen (eds) *The Routledge Handbook of Gender and Development*, The Routledge Handbook of Gender and Development (Oxford: Routledge, 2015), 560–70.

13 Elena Fiddian-Qasmiyeh and Julia Pacitto, ‘Writing the Other into Humanitarianism: A Conversation between “South-South” and “Faith Based” Humanitarianisms’, in Zeynep Sezgin and Dennis Dijkzuel (eds) *The New Humanitarians in International Practice* (London: Routledge, 2015), 285.

14 Brković, *Realigning Humanitarianism,* 72–74; Čarna Brković, ‘Socialist Modernist Worldmaking: Yugoslav Interventions in the International Humanitarian Debates in the 1970s’, *Humanity: An International Journal of Human Rights, Humanitarianism, and Development,* 15, 1 (2024), 25; Siobhán Hearne, ‘Between Moscow and Geneva: The Soviet Red Cross in the International Red Cross Movement’, *Journal of Global History*, 20, 1 (2025), 66.

15 Oscar A. Gómez, ‘Localisation of Deglobalisation? East Asia and the Dismantling of Liberal Humanitarianism’, *Third World Quarterly,* 42, 6 (2021), 1353–54. The Soviet Red Cross refused foreign aid in the immediate aftermath of the Chernobyl disaster, International Federation of the Red Cross Archives, box R510345567, USSR, Alliance of Red Cross and Red Crescent Societies (1987–1989). Telex from Soviet Red Cross, 5 May 1986.

16 Dora Tot, ‘Translating Humanitarian Aid into Technical Cooperation: Yugoslavia's Medical Assistance to the Algerian National Liberation Movement (1958–1965)’, *International History Review*, 46, 6 (2024), 769; Severyan Dyakonov, ‘“Resilience, Perseverance, and Sense of Diplomacy” The Soviet Red Cross in India, 1954–1963’, *European Journal for the History of Medicine and Health,* 82 (2025), 75. See also Bogdan Iacob's article in this special issue.

17 Miwa Hirono, ‘Three Legacies of Humanitarianism in China’, *Disasters*, 37, S2 (2013), 5208; Jack Neubauer, ‘Adopting Revolution: The Chinese Communist Revolution and the Global Politics of Humanitarianism’, *Modern China,* 47, 5 (2020), 606–7.

18 P. Sean Brotherton, ‘Social Medicine, Otherwise: Cuban Health(Care) as Political Praxis’, in Anne Kveim Lie, Jeremy A. Greene, and Warwick Anderson (eds) *Medicine on a Larger Scale: Global Histories of Social Medicine* (Cambridge: Cambridge University Press, 2025), 307–8.

19 Robert Huish, ‘Why Does Cuba “Care” So Much? Understanding the Epistemology of Solidarity in Global Health Outreach’, *Public Health Ethics,* 7, 3 (2014), 272.

20 Ljubica Spaskovska, ‘Constructing the “City of International Solidarity”: Non-Aligned Internationalism, the United Nations and Visions of Development, Modernism and Solidarity, 1955–1975’, *Journal of World History,* 31, 1 (2020), 137–64; Luminita Gatejel, ‘Bargaining from Humanitarian Aid Across the Iron Curtain: Western Relief Workers in Romania in the Late 1970s’, *Cold War History*, 22, 1 (2022), 41–57; Cristian Capotescu, ‘Migrants into Humanitarians: Ethnic Solidarity and Private Aid-Giving during Romania's Historic Flood of 1970’, *East European Politics and Societies*, 35, 2 (2020), 293–312; Alexander Poster, ‘The Gentle War: Famine Relief, Politics, and Privatization in Ethiopia, 1983–1986’, *Diplomatic History*, 36, 2 (2012), 399–425; Katja Doose, ‘Tremor and Change: Humanitarian Interventions after the 1988 Earthquake in Armenia’, in Jo Laycock and Francesca Piana (eds) *Aid to Armenia: Humanitarianism and Intervention from the 1890s until the Present* (Manchester: Manchester University Press, 2020), 131–46.

21 Boyd van Dijk, ‘“The Great Humanitarian”: The Soviet Union, the International Committee of the Red Cross, and the Geneva Conventions of 1949’, *Law and History Review*, 37, 1 (2019), 209–35; Raluca Grosescu and Ned Richardson-Little, ‘Revisiting State Socialist Approaches to International Criminal and Humanitarian Law: An Introduction’, *Journal of the History of International Law*, 21, 2 (2019), 161–80; Giovanni Mantilla, ‘The Protagonism of the USSR and Socialist States in the Revision of International Humanitarian Law’, *Journal of the History of International Law*, 21, 2 (2019), 181–211.

22 Bogdan C. Iacob, ‘Health as a Human Right and Eastern European Anticolonialism’, in Raluca Grosescu and Ned Richardson-Little (eds) *Socialism and International Law: The Cold War and its Legacies* (Oxford: Oxford University Press, 2024), 137–62.

23 Brković, *Realigning Humanitarianism,* 74–80.

24 Dora Tot, ‘Translating Humanitarian Aid into Technical Cooperation’, 772–73; Hearne, ‘Between Moscow and Geneva’; Dora Vargha, ‘The Socialist World in Global Polio Eradication’, *Revue d’études comparatives Est-Ouest*, 1, 1 (2018), 71–94; Bogdan C. Iacob, ‘Malariology and Decolonisation: Eastern European Experts from the League of Nations to the World Health Organisation’, *Journal of Global History*, 17, 2 (2022): 233–53; Brković, *Realigning Humanitarianism*, 85.

25 Peter Whitewood, ‘“A Few Regrettable Cases”: Civil War Violence and the Recognition of the Russian Red Cross Society, 1918–21’, *International History Review,* (2024), 2; Jo Laycock, ‘Saving the Remnant or Building Socialism? Transnational Humanitarian Relief in Early Soviet Armenia’, *Moving the Social,* 57 (2017), 87; Hanna Matt, ‘Reconstituting Relief: Famine and the Russian Red Cross in Post-Revolutionary Central Asia (1921–1923)’, in *The Red Cross Facing the Red Star: Humanitarianism and Communism in the XXth Century,* 95–108.

26 Michael G. Panzer, ‘Building a Revolutionary Constituency: Mozambican Refugees and the Development of the FRELIMO Proto-state, 1964–1968’, *Social Dynamics,* 39, 1 (2013), 5–23; Ana Guardião ‘Political Beneficiaries of Humanitarianism? The FNLA and the Angolan Refugee Crisis in the Congo (1960–1975)’, *Cold War History* (2025); Marie Cugnet, ‘Les Croix-Rouge vietnamiennes communistes: de la co-construction étatique à l’insertion dans le Mouvement Croix-Rouge (1946–1976)’, in *The Red Cross Facing the Red Star,* 246–265. See also Alila Brossard Antonielli's article in this special issue.

27 Iacob, ‘Paradoxes of Socialist Solidarity’, 118. See also Vargha, ‘Technical Assistance’.

28 Brković, ‘Socialist Modernity Worldmaking’, 33.

29 Young-sun Hong, *Cold War Germany, the Third World, and the Global Humanitarian Regime* (Cambridge: Cambridge University Press, 2015); Arianna Pasqualini, ‘Health, Solidarity, and Propaganda under Real Socialism: East German Medical Aid to SWAPO’, *European Journal for the History of Medicine and Health,* 82 (2025), 135–62.

30 Maria Framke, ‘Political Humanitarianism in the 1930s: Indian Aid for Republican Spain’, *European Review of History,* 23, 1–2 (2016), 63–81; Laurence Brown, ‘The Great Betrayal? European Socialist and Humanitarian Relief during the Spanish Civil War’, *Labour History Review*, 67, 1 (2002), 83–99; Eric R. Smith, *American Relief Aid and the Spanish Civil War* (Columbia: University of Missouri Press, 2013); Salar Mohandesi, *Red Internationalism: Anti-Imperialism and Human Rights in the Global Sixties and Seventies* (Cambridge: Cambridge University Press, 2023).

31 Jessica Dalljo, ‘“Solidarity is a Matter of the Heart”: Anti-Imperialist Solidarity Donations in GDR Children's Magazines’, *International Review of Social History,* 69, 32 (2024), 159–76; Maren Hachmeister, ‘“Without Solidarity, No People”: International Solidarity in the East German People's Solidarity’, *International Review of Social History*, 69, 23 (2024), 117–37; Nikola Tohma and Julia Reinke, ‘““Like we would help brothers or sisters”? Practising Solidarity with Greek Civil War Refugees in Socialist Czechoslovakia and the GDR in the Shadow of World War II’, *International Review of Social History,* 69, 32 (2024), 13–41; James Mark et al. ‘“We Are with You, Vietnam”: Transnational Solidarities in Socialist Hungary, Poland and Yugoslavia’, *Journal of Contemporary History,* 50, 3 (2015), 439–64; Idesbald Goddeeris, ‘Between Anti-Imperialism and Anti-Communism: Poland and International Solidarity with Vietnam’, in Alexander Sedlmaier (eds) *Protest in the Vietnam War Era* (Cham: Palgrave, 2022), 113–39.

32 Péter Apor and James Mark, ‘Home Front’, in *Socialism Goes Global,* 318–58.

33 George Bodie, ‘Introduction to Everyday Internationalism: Socialist–South Connections and Mass Culture during the Cold War’, *International Review of Social History,* 69, S32 (2024), 8.

34 Maren Hachmeister, ‘Self-organised Care for Older People in East Germany: Local Humanitarianism and Post-1989 Transformations’, in *Humanitarian Mobilization in Central and Eastern Europe,* 94–112.

35 Brotherton, ‘Social Medicine, *Otherwise*’, 298.

36 Berthold Unfried and Claudia Martínez Hernández, ‘Cuban *Internacionalismo*: A Cuban Contribution to the History of Internationalisms’, in Su Lin Lewis and Nana Osei-Opare (eds) *Socialism, Internationalism, and Development in the Third World: Envisioning Modernity in the Era of Decolonization* (London: Bloomsbury Academic, 2024), 140–41.

37 Alila Brossard Antonielli, ‘Medical *Cooperantes* in Mozambique, Adapting and Contributing to a Socialist Primary Health Care 1970–1985’, *European Journal for the History of Medicine and Health,* 82 (2025), 162–99; Eric Burton, ‘Frontline Citizens: Liberation Movements, Transnational Solidarity, and the Making of Anti-Imperialist Citizenship in Tanzania’, *International Review of Social History,* 69, S32 (2024), 218–20.

38 ‘Milliony dongov – Armenii’ *Sovetskii Krasnyi Krest,* 6 (1989), 3; L. Ryl'skaia, ‘Beda otozvalas’ v kazhdom serdtse’, *Sovetskii Krasnyi Krest*, 2 (1989), 7.

39 Francesca Rolandi, ‘The 1976 Friuli Earthquake and Yugoslav Aid: Practicing Solidarity, Reframing Hierarchies, and Looking for New Markets through Humanitarianism’, *Contemporanea,* 27, 2 (2024), 302–7; Cristian Capotescu, ‘When the “Socialist Good Life” Met Its Match: Austerity and Humanitarian Crisis in 1980s Romania’, in *Humanitarian Mobilization in Central and Eastern Europe,* 75–93.

40 For an overview, see Iacob, ‘Health’ in *Socialism Goes Global,* 390–6.

41 Capotescu, ‘When the “Socialist Good Life” Met Its Match’, 86–8.

42 Brković, ‘Socialist Modernist Worldmaking’, 19.

43 James Mark, Anikó Imre, Bogdan C. Iacob, and Catherine Baker, ‘Introduction: Racial Disavowals – Historicising Whiteness in Central and Eastern Europe’ in James Mark, Anikó Imre, Bogdan C. Iacob, and Catherine Baker (eds) *Off White: Central and Eastern Europe and the Global History of Race* (Manchester: Manchester University Press, 2024), 11.

44 Gregory Witkowski, ‘Between Fighters and Beggars: Socialist Philanthropy and the Imagery of Solidarity in East Germany’, in Quinn Slobodian (eds) *Comrades of Colour: East Germany in the Cold War World* (New York: Berghahn, 2015), 73–94.

45 Dongxin Zou's work on Chinese medical assistance to Algeria in the 1960s details how Chinese medical teams had to overcome the bias of local populations who regarded European physicians as superior, Dongxin Zou, ‘Socialist Medicine and Maoist Humanitarianism: Chinese Medical Missions to Algeria, 1963–1984’, PhD thesis (Columbia University, 2019), 143.

46 Jeffrey J. Byrne, *Mecca of Revolution: Algeria, Decolonization, and the Third World Order* (New York: Oxford University Press, 2016), 134–139; George Roberts, *Revolutionary State-Making in Dar es Salaam: African Liberation and the Global Cold War, 1961*–*1974* (Cambridge: Cambridge University Press, 2022), chapter 3; Eline van Ommen, *Nicaragua Must Survive: Sandinista Revolutionary Diplomacy in the Global Cold War* (Oakland: University of California Press, 2024). For a study focused specifically on socialist aid, see Thi-Hong Luong and Ha Hai Hoang, ‘Dancing with the Wind: The Diplomacy of Communist Bloc Economic Aid to North Vietnam, 1954–1975’, *The Historian,* 87, 1 (2025), 44–65. For a non-socialist context, see David C. Engerman, *The Price of Aid: The Economic Cold War in India* (Cambridge: Harvard University Press, 2018).

